# Neuropathic-like pain features and cross-sectional associations in rheumatoid arthritis

**DOI:** 10.1186/s13075-015-0761-8

**Published:** 2015-09-03

**Authors:** Sanne M. W. Koop, Peter M. ten Klooster, Harald E. Vonkeman, Laura M. M. Steunebrink, Mart A. F. J. van de Laar

**Affiliations:** Department of Internal Medicine, Hospital Group Twente, Almelo, The Netherlands; Arthritis Center Twente, Department of Psychology, Health and Technology, University of Twente, Enschede, The Netherlands; Arthritis Center Twente, Department of Rheumatology and Clinical Immunology, Medical Spectrum Twente, Enschede, The Netherlands

## Abstract

**Introduction:**

Increasing evidence indicates that features suggestive of neuropathic pain may also be present in patients with common rheumatic conditions. The objective of this study was to examine neuropathic-like pain symptoms and associated factors in patients with rheumatoid arthritis.

**Methods:**

We used the painDETECT screening tool to identify possible or likely neuropathic pain in 159 outpatients with rheumatoid arthritis. Patients additionally completed other self-reported measures, while clinical measures were assessed to calculate the 28-joint Disease Activity Score. Univariate analyses and multivariable logistic regression were used to identify factors associated with neuropathic pain features.

**Results:**

According to the painDETECT, 27 patients (17.0 %) were classified as having likely neuropathic pain and 34 patients (21.4 %) as having possible neuropathic pain. Besides reporting more severe pain, patients with likely or possible neuropathic pain were more likely to meet the diagnostic criteria for fibromyalgia, to use analgesics, and to have more tender joints and a worse physical and mental health status as measured by the 36-item Short-Form health survey. In multivariable analysis, physical (*P* < 0.001) and mental health status (*P* = 0.006) remained significantly associated with neuropathic pain features, even after controlling for pain severity.

**Conclusions:**

These findings suggest that a sizeable proportion of patients with relatively well-controlled rheumatoid arthritis report symptoms suggestive of neuropathic pain. Neuropathic-like pain symptoms are independently associated with worse self-reported physical and mental health.

## Introduction

Rheumatoid arthritis (RA) is the most common type of autoimmune arthritis. For patients with RA, pain is the predominant impairment and most important priority for improvement [1]. RA pain is traditionally attributed to peripheral inflammation of the joints [2]. In recent years much progress has been made in the treatment of RA and especially in reducing this inflammation. With the help of combinations of disease-modifying antirheumatic drugs (DMARDs) and biologic therapies, an increasing number of RA patients now experience extensive periods of low disease activity. Some patients even reach sustained remission. However, pain control is often inadequate and significant pain persists in a substantial proportion of patients, even when inflammation appears to be well controlled [3].

This suggests that inflammation or subsequent joint damage might not be the only factor causing pain in RA. Although pain in RA is often described as ‘gnawing’ or ‘aching’, descriptors that have typically been associated with nociceptive pain, some RA patients also use typical neuropathic pain (NP) qualities such as ‘burning’ or ‘prickling’ [4–6]. NP is assumed to be caused by a lesion or disease affecting the somatosensory nervous system [7]. It is maladaptive and persists in the absence of noxious stimuli and no, or minimal, peripheral inflammatory pathology [8]. NP symptoms include several abnormal sensations, including hyperalgesia and allodynia.

Although there is generally no apparent lesion of the nervous system, accumulating evidence suggests that symptoms of NP may also be present in patients with rheumatic conditions such as fibromyalgia (FM) or osteoarthritis. Prevalence estimates of NP-like features have ranged from around 30 % in osteoarthritis [9–11] to 50–75 % in FM [12, 13]. Recently, Wu et al. [14] also demonstrated a NP component in ankylosing spondylitis, a disease regarded as prototypical of inflammatory pain. A mixed pain concept, in which different pain mechanisms operate, may also be applicable to RA. As NP does not respond consistently to traditional anti-inflammatory RA medications [15], and may be associated with detrimental quality of life [10, 16, 17], it is important to explore the presence of NP symptoms in RA.

Screening tools based on verbal pain descriptors and pain qualities are frequently used to distinguish NP from other types of chronic pain [18]. One of these measures is the painDETECT [19], a self-report questionnaire with nine items that does not require a clinical examination. To date, few studies have specifically examined the prevalence of NP features in RA. Only three studies in (subsamples of) RA patients estimated the prevalence of NP using screening tools such as the painDETECT [20–22]. However, prevalence rates of likely NP varied widely between 5 % and 36 %. Moreover, in addition to the small sample sizes involved, none of these studies investigated multivariable associations with sociodemographic and clinical factors. Therefore, the objectives of this study were to examine the occurrence of neuropathic-like pain symptoms in patients with RA using the painDETECT questionnaire and to investigate possible associated factors.

## Methods

### Study population and data collection

Data for this study were collected within the Dutch Rheumatoid Arthritis Monitoring (DREAM) registry. The DREAM registry is a prospective, multicenter initiative to monitor the course of RA patients in the Netherlands. Both patient-reported and clinical outcomes are collected and monitored using a web-based data acquisition and storage system. Patient-reported outcomes are generally completed preceding each visit to the outpatient clinic. Between June 2013 and April 2014, RA patients from the Medisch Spectrum Twente hospital participating in the DREAM registry were informed about the study and invited to participate upon logging on to their patient portal. According to the Dutch Medical Research Involving Human Subjects Act, the study did not need approval of the ethical review board as only (nonintervention) studies with a high burden for patients have to be reviewed. All DREAM patients have provided written informed consent to participate in the registry. Patients were fully informed about the nature of this additional survey study and the voluntary nature was emphasized.

### Measures

Patients completed a series of standardized questionnaires. Additional information was gathered on sociodemographics, disease duration, self-reported pain medication, and smoking and alcohol use.

#### The painDETECT

NP symptoms were assessed using the Dutch version of the painDETECT [23]. The painDETECT consists of seven items evaluating pain qualities, one evaluating the course pattern of pain, and one evaluating pain radiation. Additionally, the questionnaire contains three 0–10 numerical rating scales (NRSs) for current, worst, and average pain severity. An overall score is generated that summarizes everything but the pain intensity NRSs, which ranges between −1 and 38. An overall score >18 indicates likely NP, 13–18 possible NP, and <13 unlikely NP [19].

#### The Fibromyalgia Survey Questionnaire

The Fibromyalgia Survey Questionnaire (FSQ) was used to assess whether the RA patients met the modified ACR 2010 diagnostic criteria for FM [24]. The FSQ consists of the widespread pain index (WPI) of 19 areas of the body and a symptom severity score (SSS) addressing fatigue, waking unrefreshed, and cognitive symptoms together with further somatic symptoms. Patients satisfy the FM survey criteria if WPI ≥7 and SSS ≥5 or when WPI between 3–6 and SSS ≥9.

#### The Health Assessment Questionnaire disability index

Physical disability was assessed with the Health Assessment Questionnaire disability index (HAQ-DI) which contains 20 items measuring physical disabilities over the past week in eight categories of daily living [25]. Items are scored from 0 (without any difficulty) to 3 (unable to do). A total score is calculated by averaging the highest score in each category (corrected for the use of aids and devices) if at least six categories are completed.

#### The SF-36v2 health survey

Health status was measured using the 36-item Short-Form (SF-36) Health Survey version 2 (SF-36v2) which assesses eight different aspects of health [26]. Item scores can be aggregated into a physical component summary (PCS) and mental component summary (MCS) score. The component summary scores are standardized using normative data from the 1998 US general population with a mean score of 50 and a standard deviation (SD) of 10.

#### Clinical assessment of disease activity

Clinical data were collected during the patient’s visit to the outpatient clinic, including a 28-tender joint count (TJC), 28-swollen joint count (SJC), the erythrocyte sedimentation rate (ESR) and C-reactive protein (CRP). The TJC and SJC examinations were conducted by a trained rheumatology nurse. The TJC, SJC, and ESR were used to compute the CRP, C-reactive protein (DAS28). DAS28 scores range between 0 and 10, with a score <2.6 indicating clinical remission and ≤3.2 representing low disease activity [27].

### Statistical analyses

Patients with possible or likely NP symptoms (painDETECT ≥13) were compared to patients without NP symptoms. Univariate differences were examined using independent *t*-tests for normally distributed variables, Mann–Whitney *U* tests for non-normally distributed variables, and chi-square tests (with Yates’ continuity correction as appropriate) for categorical variables. Variables with a marginal significance (*P* ≤ 0.20) in the univariate analyses were included in a multivariable binary logistic regression model to identify unique associations with NP symptoms. To avoid multicollinearity problems, Pearson correlations were calculated between the independent variables. In case of highly related variables (*r* > 0.5), only one variable was considered for the initial logistic regression model. Final models were additionally controlled for current, worst, and average pain severity. Multivariable models were tested for goodness of fit with the Hosmer and Lemeshow test. The explained variance was examined using Nagelkerke’s pseudo *R*^2^. Statistical significance was defined as *P* ≤ 0.05 (two-tailed). All analyses were performed with SPSS 22 (SPSS Inc., Chicago, IL).

## Results

### Patient characteristics

In total, 174 patients completed the painDETECT before their visit to the outpatient clinic. Complete medical and clinical data were available for 159 patients. Most patients had well-controlled disease activity (Table 1). Of the 159 patients, 119 (74.8 %) were in remission (DAS28 < 2.6) and another 21 (13.2 %) had low disease activity (DAS28 2.6–3.2). The majority of patients were being treated with a DMARD (or DMARD plus prednisolone) and/or a biologic agent at the time of the study. A total of 101 patients (63.5 %) were prescribed nonsteroidal anti-inflammatory drugs (NSAIDs) continuously or on-demand, while only 4 (2.5 %) were prescribed nonNSAID analgesics.

**Table 1:** Patient characteristics (*n* = 159)

Female sex, n (%)	104 (65.4)
Age (years), mean ± SD	57.2 ± 10.9
Education, n (%)	
Low	37/158 (23.4)
Medium	93/158 (58.9)
High	28/158 (17.7)
Current smoking (yes), n (%)	27 (17.0)
Current alcohol use (yes), n (%)	120 (75.5)
Disease duration (years), median (IQR)	6.0 (3.0–12.0)
Concomitant FM (yes), n (%)	23/158 (14.6)
Prescribed RA medication	
DMARD	95 (59.7)
DMARD plus biologic	40 (25.2)
Biologic only	18 (11.3)
NSAID only	5 (3.1)
Other painkiller only	1 (0.6)
Self-reported current pain medication (yes), n (%)	81/158 (51.3)
Self-reported current neuropathic pain medication (yes), n (%)	9/158 (5.7)
DAS28 (0–10), mean ± SD	2.1 ± 1.0
ESR (mm/hour), median (IQR)	8.0 (5.0–17.0)
CRP (mg/l), median (IQR)	3.0 (1.0–7.0)
SJC (0–28), median (IQR)	0 (0–1)
TJC (0–28), median (IQR)	0 (0–1)
HAQ-DI (0–3), mean ± SD	0.7 ± 0.6
SF-36 PCS (0–100), mean ± SD	41.7 ± 9.4
SF-36 MCS (0–100), mean ± SD	50.3 ± 10.3

*CRP* C-reactive protein, *DAS28* 28-joint Disease Activity Score, *DMARD* disease-modifying antirheumatic drug, *ESR* erythrocyte sedimentation rate, *FM* fibromyalgia, *HAQ-DI* Health Assessment Questionnaire disability index (standard scoring), *IQR* interquartile range, *MCS* mental component summary, *NSAID* nonsteroidal anti-inflammatory drug, *PCS* physical component summary, *RA* rheumatoid arthritis, *SF-36* 36-item Short-Form health survey, *SJC* swollen joint count, *TJC* tender joint count

### Neuropathic pain features

Despite relatively low disease activity, 70 patients (44 %) reported clinically significant pain in the past 4 weeks (average pain score ≥4 on the 10-point NRS) on the painDETECT (Table 2). According to the painDETECT scores, 17.0 % (exact 95 % confidence interval (CI) 11.5–23.7) of the patients had likely NP and 21.4 % (exact 95 % CI 15.3–28.6) had possible NP features. Most common pain qualities mentioned were pain attacks like electric shocks (34.6 %) and pain with slight pressure (45.3 %), but other clinically relevant somatosensory symptoms such as burning and prickling pain were also reported by approximately 25 % of patients. More than one-third of patients experienced radiating pain. Total painDETECT scores correlated moderately with pain severity, ranging from *r* = 0.51 for current pain to *r* = 0.56 for strongest pain (*P* < 0.001 for both).

**Table 2:** Pain intensity and sensory symptoms (painDETECT)

Etiology, mean ± SD	
Current pain (0–10)	3.3 ± 2.4
Strongest pain (0–10)	4.3 ± 2.9
Average pain (0–10)	3.5 ± 2.4
Clinically relevant complaint (score >3), n (%)	
Q1, burning	38 (23.9)
Q2, prickling	39 (24.5)
Q3, allodynia	27 (17.0)
Q4, attacks	55 (34.6)
Q5, thermal	21 (13.2)
Q6, numbness	39 (24.5)
Q7, pressure	72 (45.3)
Patterns	
Persistent pain with slight fluctuations	70 (44.0)
Persistent pain with pain attacks	15 (9.4)
Pain attacks without pain between them	57 (35.8)
Pain attacks with pain between them	17 (10.7)
Radiating pain (yes), n (%)	58 (36.5)
PainDETECT total score (0–38), mean ± SD	10.5 ± 6.8
Neuropathic pain, n (%)	4.8 ± 1.5
Unlikely (0–12)	98 (61.6)
Possible (13–18)	34 (21.4)
Likely (19–38)	27 (17.0)

### Bivariate associations with neuropathic pain features

Patients with possible or likely NP symptoms did not significantly differ from those with no NP symptoms on any of the measured sociodemographic characteristics (Table 3). Prescribed DMARD and biologic therapy was almost identical for both groups. Although the proportion of patients that were prescribed continuous or on-demand NSAIDs was also very similar in the non-NP and NP group (62.2 % versus 65.6 %, respectively; *P* = 0.67), NP patients were almost twice as likely to report actual current use of paracetamol and NSAIDs. Additionally, significantly more patients with NP symptoms also met the criteria for FM. Besides experiencing more severe pain, patients with NP symptoms reported more disability on the HAQ-DI and worse physical and mental health on the SF-36. Finally, patients with NP had significantly higher tender joint scores and total DAS28 scores therefore tended to be somewhat higher in the NP group.

**Table 3:** Comparison of patients with (painDETECT ≥13) and without neuropathic pain symptoms

	No NP	NP	*P*
	Symptoms	Symptoms	
Female sex, n (%)	61 (62.2)	43 (70.5)	0.288
Age (years), mean ± SD	57.7 (10.9)	56.3 (11.0)	0.432
Education, n (%)			
Low	22 (22.4)	15 (25.0)	
Medium	57 (58.2)	36 (60.0)	
High	19 (19.4)	9 (15)	0.768
Current smoking, n (%)	15 (15.3)	12 (19.7)	0.476
Current alcohol, n (%)	73 (74.5)	47 (77.0)	0.715
Disease duration (years), median (IQR)	6.0 (3.0–12.0)	6.0 (3.0–12.0)	0.912
FM, n (%)	6 (6.1)	17 (28.3)	<0.001
Prescribed RA medication, n (%)			
DMARD	58 (59.2)	37 (60.7)	0.854
DMARD plus biologic	25 (25.5)	15 (24.6)	0.897
Biologic	11 (11.2)	7 (11.5)	0.961
NSAID only	3 (3.1)	2 (3.3)	1.000
Self-reported current pain medication, n (%)	37 (37.8)	44 (73.3)	<0.001
Self-reported current neuropathic pain medication, n (%)	4 (4.1)	5 (8.3)	0.263
Current pain (0–10), mean ± SD	2.5 (2.2)	4.6 (2.3)	<0.001
Strongest pain (0–10), mean ± SD	3.2 (2.6)	6.0 (2.3)	<0.001
Average pain (0–10), mean ± SD	2.7 (2.2)	4.8 (2.1)	<0.001
DAS28 (0–10), mean ± SD	2.0 (1.0)	2.3 (1.0)	0.095
ESR (mm/hour), median (IQR)	8.0 (2.5–15.0)	8.0 (5.0–17.0)	0.610
CRP (mg/l), median (IQR)	2.0 (1.0–7.0)	3.0 (2.0–8.0)	0.107
SJC (0–28), mean ± SD	0 (0–1)	0 (0–1)	0.603
TJC (0–28), mean ± SD	0 (0–1)	0 (0–2)	0.016
HAQ-DI (0–3), mean ± SD	0.5 (0.5)	1.1 (0.6)	<0.001
SF-36 PCS (0–100), mean ± SD	44.9 (8.2)	36.4 (8.7)	<0.001
SF-36 MCS (0–100), mean ± SD	52.7 (9.4)	46.5 (10.6)	<0.001

*CRP* C-reactive protein, *DAS28* 28-joint Disease Activity Score, *DMARD* disease-modifying antirheumatic drug, *ESR* erythrocyte sedimentation rate, *FM* fibromyalgia, *HAQ-DI* Health Assessment Questionnaire disability index (standard scoring), *IQR* interquartile range, *MCS* mental component summary, *NP* neuropathic pain, *NSAID* nonsteroidal anti-inflammatory drug, *PCS* physical component summary, *RA* rheumatoid arthritis, *SF-36* 36-item Short-Form health survey, *SJC* swollen joint count, *TJC* tender joint count

### Multivariable associations with neuropathic pain features

Since current, average and strongest pain were strongly correlated with pain medication use (*r* = 0.49–0.53), HAQ-DI scores (*r* = 0.64–0.66), and PCS scores (*r* = 0.67–0.69), pain severity was not included in the initial multivariable model. Additionally, the HAQ-DI and PCS (*r* = −0.75) and the total DAS28 and TJC28 (*r* = 0.58) correlated strongly with each other. As the TJC28 was more strongly associated with NP in univariate analysis than the total DAS28, it was selected for inclusion in the multivariable model. Although the HAQ-DI was slightly more strongly associated with NP than the PCS, the latter was selected over the HAQ-DI as it provides a more comprehensive assessment of physical health status than physical disabilities alone. The initial model without pain severity as a covariate (Table 4) showed a good fit to the data (Hosmer and Lemeshow test: *χ*^2^(8) = 2.215, *P* = 0.974). NP symptoms were independently associated with worse physical and mental health status. Meeting the FM criteria, self-reported pain medication, CRP values and number of tender joints were not associated with NP symptoms in multivariable analysis. Controlling for current pain severity in the final model (Hosmer and Lemeshow test: *χ*^2^(8) = 4.139, *P* = 0.844) did not substantially alter the odds ratios nor the significance of any of the associations. Additional models controlling for strongest or average pain severity instead of current pain severity yielded very similar results.

**Table 4:** Multivariable associations with neuropathic pain symptoms (painDETECT ≥13)

	Initial model		Final model	
	OR (95 % CI)	*P*	OR (95 % CI)	*P*
Current pain	–	–	1.08 (0.86–1.35)	0.525
FM	1.73 (0.53–5.60)	0.360	1.78 (0.54–5.67)	0.350
Self-reported current pain medication	2.24 (0.98–5.15)	0.056	2.06 (0.87–4.92)	0.102
CRP	0.99 (0.94–1.05)	0.816	0.99 (0.94–1.04)	0.795
Number of TJC	0.96 (0.84–1.09)	0.508	0.95 (0.83–1.08)	0.434
SF-36 PCS	0.90 (0.86–0.95)	<0.001	0.91 (0.86–0.97)	0.003
SF-36 MCS	0.96 (0.92–0.99)	0.021	0.96 (0.92–1.00)	0.046

Nagelkerke *R* ^2^ = 0.36 for both models

*CI* confidence interval, *CRP* C-reactive protein, *FM* fibromyalgia, *MCS* mental component summary, *OR* odds ratio, *PCS* physical component summary, *SF-36* 36-item Short-Form health survey, *TJC* tender joint count

## Discussion

This study assessed the occurrence and associations of NP-like symptoms in a cohort of patients with relatively well-controlled RA. Despite almost 75 % of patients being in DAS28 remission, 44 % still reported clinically significant pain. According to the PainDETECT, 17 % of all patients had likely NP, while 21 % had possible NP features. These patients were shown to have more severe pain, used pain medication more often, and reported lower quality of life. Multivariable logistic analysis showed that the occurrence of possible or likely NP-like features was independently associated with worse physical and mental health, even when controlling for pain severity.

Despite the relatively low disease activity in the current cohort, nearly half of the patients still reported clinically significant pain. Clinically significant pain was defined as an average pain score ≥4. This threshold was based on several previous studies that identified pain intensity levels ≥4 out of 10 as moderate to severe or unacceptable [28, 29]. This confirms that, despite improvements in the management of RA, pain control remains inadequate in some patients, even when inflammation is well controlled [3]. Consequently, although inflammation contributes to pain in RA, it may not be the only factor and other pain mechanisms such as NP may also play a role.

The findings of this study suggest that the pain of a substantial number of RA patients may have neuropathic features or a neuropathic component, as has also been shown in other rheumatic conditions. NP symptoms have only been sporadically tested in RA samples. The 17 % proportion of RA patients with likely NP found in this study was very similar to the 19 % prevalence reported by Meirinhos et al. [21]. Conversely, Ahmed et al. [20] reported that 28 % of RA patients had possible NP symptoms and 5 % were likely to be experiencing NP symptoms according to the painDETECT. The relatively high proportion of patients (36 %) with NP in the study by Perrot et al. [22] may have resulted from their use of the DN4 questionnaire, which tends to have a high sensitivity but low specificity in identifying NP [30].

With respect to possible NP symptoms in patients with RA, it should be noted that although inflammatory pain and NP are attributed to different mechanisms, they do have some features in common which may confound the results of NP screeners. Most notably, the painDETECT item dealing with pain with slight pressure may also reflect typical inflammatory joint pain. Removing this item from the total painDETECT score lowered the proportion of patients with possible NP from 38.4 % to 29 % in the current study.

The proportion of RA patients with likely NP was clearly lower than that usually reported in patients with osteoarthritis [9–11] and much lower than that found in patients with FM [12, 13]. Many recent studies have suggested the NP-like symptoms in rheumatic conditions to be manifestations of dysregulation of central pain processing mechanisms [8, 12, 31–38]. Central sensitization is the increased responsiveness of nociceptive neurons in the central nervous system to normal input and the recruitment of a response to normally subthreshold inputs [39]. The precise mechanisms behind this pain augmentation are not known as yet, but most likely encompasses multiple changes in the central nervous system. In acute pain, sensitization is a physiological process to induce the body to protect damaged tissue and to give it time to heal and is thus an important mechanism for survival. It is hypothesized that in chronic pain conditions this adaptive pain mechanism persists despite elimination of the original nociceptive input and thus provides a deranged pathological mechanism for persistent pain [40]. The finding that the number of RA patients concurrently fulfilling the current criteria for FM, often considered the prototypical central pain syndrome [41], was substantially higher among those with NP features provides support to this theory for RA patients as well.

No previous studies have thoroughly examined multivariable associations of NP symptoms with sociodemographic, clinical, and quality of life-related factors in patients with RA. In the current study, NP symptoms were not related to any of the sociodemographic characteristics included. Moreover, while the TJC was significantly higher in the NP group, the more objective parameters of disease activity, such as CRP, ESR and the SJC, did not differ between the two groups. The TJC and the global health score are largely subjective measures and thus may not necessarily reflect clinical disease activity. Previous studies have shown that some patients fail to reach RA remission criteria due to poor patient-reported health scores only [42, 43]. When comparing DAS28 scores in RA and FM patients, FM patients tend to score higher on subjective parameters, while RA patients score worse on objective measures [44]. Similarly, Ranzolin et al. [45] found that RA patients with co-existent FM had significantly higher DAS28 scores mostly caused by the TJC and general health scores. As such, this also points to other mechanisms than inflammation as a cause for pain in certain RA patients.

Finally, the finding that NP symptoms were independently associated with physical and mental health status is in accordance with studies in patients with established peripheral or central neuropathology, such as diabetic neuropathy and spinal cord injury. These studies have consistently shown that the presence and severity of NP is associated with substantial impairments in most important health-related quality of life domains [46, 47]. Studies in osteoarthritis also showed that the occurrence of NP-like symptoms was strongly correlated with a worse quality of life, more distress, and higher pain intensity [10, 17].

Taken together, the findings of the current study provide preliminary support of a noninflammatory pain component in RA. This could have important implications for RA treatment strategies. The current goal in the treatment of RA is to reach early sustained remission. This is increasingly done by intensive treat-to-target strategies, aimed at DAS28 remission criteria. When remission is not reached, drug therapy is adjusted. This can range from a dose change, starting combination therapy with a second DMARD or glucocorticoids, or the start of biologicals. These latter are expensive therapies and have, as do all medications, unwanted and in some cases serious side effects. Furthermore, all these strategies specifically target inflammation. If, however, mechanisms other than inflammation, for example hypersensitivity, contribute to a high DAS28 and therefore not reaching remission, these expensive medications will not likely have the desired effect. Overtreatment could therefore be an outcome in some RA patients. In these cases, pain treatment targeting the NP-like symptoms might be more appropriate than intensifying anti-inflammatory treatment. This treatment could comprise neuromodulators and certain antidepressants. Both these drug groups appear to be effective in NP [48]. In FM, a syndrome hypothesized to be caused by central pain processing abnormalities, these drugs have also been shown to have an effect, albeit small, on pain [49, 50]. If effective in RA patients with NP-like symptoms who fail to reach remission, these therapies could be more cost-effective and, more importantly, result in a better quality of life than current treatment strategies.

Our study has several strengths. First, it combines the painDETECT questionnaire with clinical data, the DAS28 score and measurements of FM, quality of life, and disability. This gives insight into the origin of pain in RA patients, but also the effect it has on the daily life of patients. Second, the patients included in this study were recruited from normal daily clinical practice and therefore closely resemble the current ‘well-controlled’ RA patient. The findings, however, may not be applicable to RA populations with less well-controlled disease. Furthermore, since our population consisted of predominately white patients, the findings may not be generalizable to more ethnically diverse populations.

The major limitation of the study is the absence of a gold standard for NP or central sensitization. This is a problem inherent to the field. Central sensitization especially is a relatively new concept, and objective measurements do not yet exist. The gold standard is thus usually based on a combination of quantitative sensory testing and clinical or expert opinion. Additionally, the cross-sectional nature of this study does not allow any determination of the direction of associations between NP symptoms and clinical variables. Finally, the painDETECT was developed as a screening tool for NP, not central sensitization. Although symptoms of NP and central sensitization may be similar, they are by no means the same. The occurrence of NP symptoms according to the painDETECT therefore does not measure central sensitization per se, but may be useful in assisting in the identification of central sensitization [12]. More research, however, is needed to develop validated tools for the specific identification of central sensitization.

## Conclusions

This study demonstrates that neuropathic-like pain symptoms are present in a substantial number of patients with RA and are associated with worse physical and mental health. These symptoms may represent central sensitization and underscore the need for further research and screening of pain mechanisms in RA patients.

## Abbreviations

CI: Confidence interval
CRP: C-reactive protein
DAS28: 28-joint Disease Activity Score
DMARD: Disease-modifying antirheumatic drug
DREAM: Dutch Rheumatoid Arthritis Monitoring
ESR: Erythrocyte sedimentation rate
FM: Fibromyalgia
FSQ: Fibromyalgia Survey Questionnaire
HAQ-DI: Health Assessment Questionnaire disability index
MCS: Mental component summary
NP: Neuropathic pain
NRS: Numerical rating scale
NSAID: Nonsteroidal anti-inflammatory drug
PCS: Physical component summary
RA: Rheumatoid arthritis
SD: Standard deviation
SF-36: 36-item Short Form
SF-36v2: 36-item Short-Form Health Survey version 2
SJC: Swollen joint count
SSS: Symptom severity score
TJC: Tender joint count
WPI: Widespread pain index
